# Perfusion quality does not necessarily predict ultrastructural preservation after hyperosmotic brain perfusion

**DOI:** 10.64898/2026.07.27.741074

**Published:** 2026-07-30

**Authors:** Andria Slaughter, Macy Garrood, Katelyn Hedden, Allison Sowa, William Janssen, Emma L. Thorn, Claudia De Sanctis, Kurt Farrell, John F. Crary, Andrew T. McKenzie

## Abstract

Perfusion fixation is widely used in neuroscience to prepare mammalian brain tissue for histological and ultrastructural analysis. Perfusion protocols are commonly assessed using macroscopic indicators such as gross appearance and neuroimaging, which assess the extent to which perfusate has been distributed throughout the brain. There is a critical need to determine to what extent these metrics can accurately predict high-quality ultrastructural preservation, particularly as new perfusion protocols are developed for connectomics. In this technical report, we describe evidence that these two measures can be decoupled by the addition of dehydrating agents to the perfusate solution. In three human brain donors and one canine brain donor perfused with a fixative solution containing 10% mannitol and 10% polyethylene glycol 35 kDa, macroscopic and radiological indicators of perfusion quality appeared adequate or favorable. However, electron microscopy revealed expanded extracellular space, shrunken cellular processes, and distorted cell membranes, consistent with an osmotic shock artifact resulting from severe hyperosmotic dehydration. Similar ultrastructural artifacts were observed in a canine brain donor perfused with 20% mannitol in 20% neutral buffered formalin without PEG. We compare these ultrastructural findings with findings from previously reported cases perfused with standard neutral buffered formalin without osmotic additives. These findings illustrate a risk of optimizing brain perfusion protocols designed to preserve neural circuitry based on macroscopic or radiological perfusion quality metrics alone, since these metrics can be satisfied while the ultrastructure is severely compromised.

## Full Text Availability

The license terms selected by the author(s) for this preprint version do not permit archiving in PMC. The full text is available from the preprint server.

